# 

**Published:** 1884-10

**Authors:** 


CONTENTS.

| | Page. |
|---|---|
| Brights Disease | 179 |
| Constipation | 180 |
| Entire Wheat Flour | 182 |
| Vermin Vastness | 183 |
| Beef-tea vs. True Food | 184 |
| Mineral Waters | 186 |
| Parasites And Their Pedigree | 188 |
| Life Is Worth Living | 189 |
| Among The Lepers | 190 |
| Poisons And Their Antidotes | 192 |
| Taking Medicines | 196 |
| Loss of Form | 197 |
| Hair Dye | 198 |
| Treatment of Eczema | 198 |

